# Seasonal bat activity related to insect emergence at three temperate lakes

**DOI:** 10.1002/ece3.3943

**Published:** 2018-03-08

**Authors:** Ioanna Salvarina, Dorian Gravier, Karl‐Otto Rothhaupt

**Affiliations:** ^1^ Limnological Insitute University of Konstanz Konstanz Germany

**Keywords:** acoustic monitoring, aquatic–terrestrial, Chiroptera, insects, season

## Abstract

Knowledge of aquatic food resources entering terrestrial systems is important for food web studies and conservation planning. Bats, among other terrestrial consumers, often profit from aquatic insect emergence and their activity might be closely related to such events. However, there is a lack of studies which monitor bat activity simultaneously with aquatic insect emergence, especially from lakes. Thus, our aim was to understand the relationship between insect emergence and bat activity, and investigate whether there is a general spatial or seasonal pattern at lakeshores. We assessed whole‐night bat activity using acoustic monitoring and caught emerging and aerial flying insects at three different lakes through three seasons. We predicted that insect availability and seasonality explain the variation in bat activity, independent of the lake size and characteristics. Spatial (between lakes) differences of bat activity were stronger than temporal (seasonal) differences. Bat activity did not always correlate to insect emergence, probably because other factors, such as habitat characteristics, or bats’ energy requirements, play an important role as well. Aerial flying insects explained bat activity better than the emerged aquatic insects in the lake with lowest insect emergence. Bats were active throughout the night with some activity peaks, and the pattern of their activity also differed among lakes and seasons. Lakes are important habitats for bats, as they support diverse bat communities and activity throughout the night and the year when bats are active. Our study highlights that there are spatial and temporal differences in bat activity and its hourly nocturnal pattern, that should be considered when investigating aquatic–terrestrial interactions or designing conservation and monitoring plans.

## 1 INTRODUCTION

Spatial or allochthonous subsidies are resources that originate in a donor habitat and enter into a food web of a recipient habitat and possibly alter its consumer‐resource dynamics (Polis, Anderson, & Holt, 1997). Aquatic insects can enter the terrestrial landscape, for example, and become a part of its food web. Bats and other terrestrial animals often feed on aquatic insects; thus, water bodies and riparian areas are important habitats for bats (reviewed in Salvarina, 2016). Water availability has been positively related to bat species richness (McCain, 2007); therefore, understanding the dependency of bats on aquatic food resources is crucial for conservation efforts. Studies that show bat dependence on aquatic resources are more numerous for rivers and streams, than for lakes (36%, 23%, and 17% studies found, respectively, in the total of papers reviewed in Salvarina, 2016); therefore, lake systems and their importance for bats need further investigation.

In Europe, all bat species are insectivorous (except of the Egyptian fruit bat in Cyprus) and many of them include aquatic insects in their diets (Vaughan, 1997). The evidence for this has been mainly from identification of prey remains in feces (reviewed by Vaughan, 1997), and to a lesser degree from stable isotopes (Lam et al., 2013), molecular analyses on feces (e.g., Krüger, Clare, Symondson, Keišs, & Pētersons, 2013), and experiments (Fukui, Murakami, Nakano, & Aoi, 2006). However, the amount of aquatic insects entering the terrestrial systems that is available to terrestrial consumers and whether this food resource fluctuates seasonally is less studied (e.g., Salvarina et al., 2017), particularly with responses to bat activity. The importance of aquatic insects as a food resource for bats may differ seasonally, for example, in early spring when other prey availability is low (Fukui et al., 2006).

Aquatic resources in many areas of the world are degrading, and further deterioration in their quality is predicted (IFRI and Veolia 2015). Additionally, climate change is expected to reduce freshwater in most dry subtropical regions (Jiménez Cisneros et al., 2014). Numerous water bodies and bat species are under conservation. All European bat species are strictly protected and listed in the Annex IV of the Council Directive 92/43/EEC 1992 on the Conservation of Natural Habitats of Wild Fauna and Flora (EC Habitats Directive 1992). The 15.8% of all Chiroptera is threatened or extinct (The IUCN Red List of Threatened Species). To set priorities in conservation policies for bats and aquatic systems and to understand better bats’ dependence on aquatic systems, it is also important to know how much bats rely on aquatic resources. Studying aquatic–terrestrial interactions is an important topic in ecology with increasing interest (e.g., Gratton, Donaldson, & Vander Zanden, 2008; Bartrons, Papes, Diebel, Gratton, & Vander Zanden, 2013) and implications, such as in helping to: (1) investigate and possibly predict the effects of climate change and eutrophication of waters on terrestrial consumers, (2) study food webs, (3) manage the conservation of ecosystems and species effectively, and (4) track transfer of contaminants from aquatic to terrestrial systems (Mogren, Walton, Parker, & Trumble, 2013).

Our general aim was to investigate aquatic subsidies into terrestrial systems and the role they play to explain the activity of bats near lakes. Therefore, we measured aquatic insect emergence from three different types of lakes located in the same region. Earlier, we showed that the total annual biomass of emerging insects from the littoral zone of a low in nutrient content lake (Lake Constance) can reach 1.789 per mg^2^/year (Salvarina & Rothhaupt, 2017). These results confirmed that a considerable insect biomass, even from small or low in production water bodies, can subsidize terrestrial consumers. We also described the pattern of insect emergence every 5 days, during three seasons (Salvarina & Rothhaupt, 2017). Here, we aimed to further investigate how these insect emergence patterns explain bat activity at multi‐temporal (nocturnally and seasonally) and spatial scales. Thus, parallel to the insect collections we monitored bat activity, using acoustic monitoring. Our main research question was as follows: Is there a general pattern of bat activity and insect emergence in all study lakes, independently of trophic condition, size and other characteristics? If so, then this pattern can be used as reference for future studies that might aim to predict indirect effects on bats due to climate change or due to modifications on aquatic systems and thus to insect emergence. To restrict other factors, we selected lakes located on same geographical and climatic region. As each lake had a unique pattern of bat activity, we further investigated what factors influence it.

As all eighteen species, reported in our broad study area, are insectivorous with varying degrees of specialization on aquatic or terrestrial insects (Hinweise LUBW 2013, Fledermausschutz Thurgau 2014, see Appendix S1) we predicted that insect availability and season will explain bat activity. We specifically hypothesized that in contrast to lakes with lower insect emergence, at lakes with higher insect emergence, bat activity will correlate stronger with aquatic insect activity than with aerial flying insects (terrestrial insects included). Thus far, studies often acoustically sample bat activity over a limited number of hours after the sunset. This approach might potentially ignore important bat activity displayed later in the night or before sunset. Another objective of our study was therefore to explore the nocturnal (throughout the night) bat activity patterns seasonally and spatially. Activity patterns of bats are suggested as a monitoring tool of animal responses to long‐term changes in climate, as it is related to climate and weather conditions (Frick et al., 2012).

## 2 MATERIALS AND METHODS

### 2.1 Study sites

**Figure 1:**
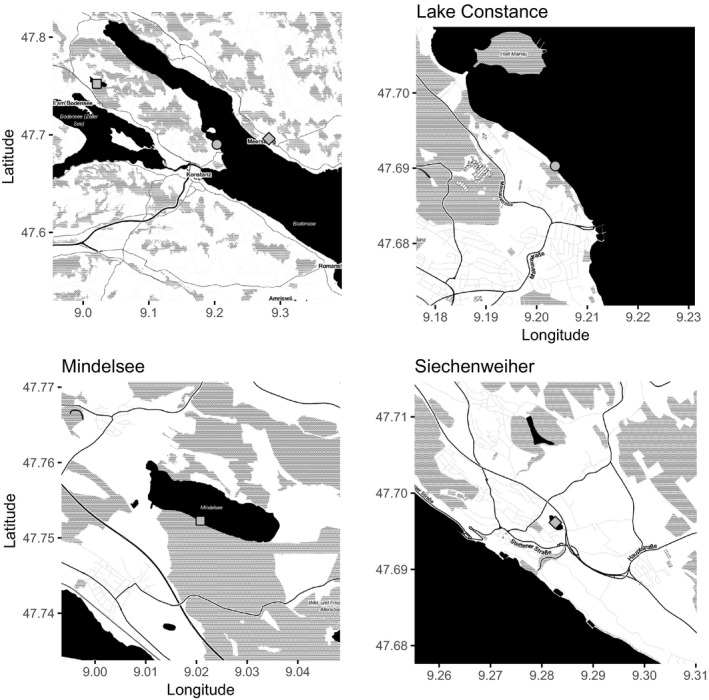
Map of the study area and all the study lakes and the sampling location in each lake: Lake Constance, Mindelsee, and Siechenweiher

The study was conducted at three lakes in South Germany that has a temperate seasonal climate (Figure 1). These three lakes (Lake Constance, Mindelsee and Siechenweiher) were chosen as representative of different nutrient content and size lakes, yet located in the same region and in the same climatic conditions. Lake Constance is a deep (max. depth 254 m), large (500 km²), prealpine, oligotrophic (low in nutrient content) lake, situated in between three countries (Germany, Switzerland, Austria). The sampling location (47^o^41′27.72″N, 9^o^12′08.18″E) was near the city of Konstanz, in Upper Lake Constance, which is less than 10 m deep in this place and is considered as a shallow area (littoral zone) (Baumgärtner, Mortl, & Rothhaupt, 2008). The shore, near our study area, was composed of forest's patches, meadows, small pastures, gardens, and orchard. Lake Mindelsee (47°45′06.95″N, 9°01′24.80″E) is a shallower (max. depth 12 m), smaller (1.02 km^2^), mesotrophic to eutrophic lake, included in a nature reserve. We sampled in the southern, steeper littoral zone which is bordered by a hill forested mainly with beech trees (Smukalla & Meyer, 1988). Siechenweiher (47^o^41′47.33″N, 9^o^16′.54.09″E) is a shallow (max. depth 2.5 m), highly eutrophic (Seenprogramm 2010), small (about 0.024 km^2^) fishing pond at the edge of the town of Meersburg. It is situated between a residential area and a busy road; however, its watershed (227 ha) is composed of forests (10%) and agricultural land (75% of which 22% is meadows, 35% arable land, and 43% orchard). Siechenweiher is about 800 m far from Lake Constance's shores, nevertheless due to its different characteristics as opposed to Lake Constance, it can be correctly considered as an independent point and not just another sampling point along the shores of Lake Constance.

We conducted fieldwork during 2 years (from July through October 2011 and from April through June 2012, plus 2 samplings in May and June 2011) to have seasons covering one “bat year” (spring, summer, autumn), when bats are active.

### 2.2 Insects

Emerging aquatic insects (hereafter referred to also as aquatic insects) were collected with floating traps (surface: 2,500 cm²: 50 × 50 cm) that looked like pyramids with a bottle of killing solution (either alcohol 80% or 1 alcohol: 1 ethylene glycol: 1 tap water) on the top. The traps were constructed at the University of Konstanz using as model similar traps used in other studies (e.g., Hagen & Sabo, 2012).

Three to five traps were placed in Lake Constance (at about 1, 2, 3, 6, 8 m, the water level varied about 175 cm), and three traps in the other lakes (at 1, 2, 5 m in Mindelsee, respectively, and at 1–1.5 m the two traps and at 2 m the third trap in Siechenweiher). The traps remained on the water from May to October 2011 and from April to June 2012. We sampled insects every 5 days, with some variation due to logistic issues (e.g., bad weather). We also collected separate insect samples the nights (from sunset till sunrise) that we recorded the bat activity. We calculated insect emergence in individuals per hour and per trap. Hereafter, we will refer to the emerged insects (per hr per trap) caught during the 5 days and nights as “total emerged insects” and to the insects emerged during bat recording nights (per hr per trap) as “night emerged insects.”

Aerial flying insects were caught using one Malaise trap constructed at the Limnological Institute, University of Konstanz (built with model the trap from Bioform^®^; 295 × 175 × 94 cm). The trap was set up at the area of the bat recordings 3 hr before the sunset (this choice was made for practical reasons, as a compromise instead of collecting insects the whole day). The first insect sample was collected at sunset. The emergence rate per hour that corresponds to these 3 hr will be referred as “day aerial insects.” The sample from sunset till sunrise per hour corresponded to the “night aerial insects.” The Malaise trap was randomly orientated to avoid bias due to wind and a possible corridor of flying insects. The aerial flying insect collection was conducted only when bats were recorded during April–June 2012.

The insect samples were counted and classified in the laboratory to order or family level (based on: Roth, 1974; Borror, Triplehorn, & Johnson, 1989; Nilsson, 1996, 1997). Usually, the Diptera were identified to family level (e.g., mainly Chironomidae, Simulidae). The emergence rate was calculated as number of individuals per trap per hr. Due to the different night length through the seasons, we preferred to express the insect emergence per hour so that it corresponds to bat activity that was also expressed per hour. For the insect emergence, the mean values from all samples/traps of the same day(s) were used for the data analysis, unless some samples were destroyed or lost.

More details on these insect collections can be found in Salvarina and Rothhaupt (2017). There, it is shown that the number of total and night emerged insects per m^2^ has a positive relationship to the biomass of day and night insect biomass, respectively. Therefore, the use of emerging insect number is sufficient. After each night insect collection, water temperature was measured about 30 min after the sunrise at a depth of about 50 cm, using a temperature/oxygen portable device (Multiline F/Set‐3, WTW Weilheim, Germany). The air temperature at the time of the sunset was taken from the online database from http://www.wunderground.com for the nearest station) for each location. Sunset air temperature and wind speed are reliable proxies for mean night temperature and wind speed, respectively. This is shown by a significant correlation between temperature and wind data for our sampling dates taken from the meteorological station in Konstanz (Data source: the German Meteorological Service (Deutsche Wetterdienst, http://www.dwd.de, assessed December 2017). The mean night temperature (from sunset to sunrise) and mean night wind speed were positively correlated to the temperature (*R* ^2^ = .92, *F*_1,60_ = 623.9, *p* < .001) and wind speed (*R* ^2^ = .63, *F*_1,60_ = 100.7, *p* < .001) at the sunset time.

### 2.3 Bats

Bat activity was assessed with acoustic monitoring during three nights (from about 20 min before the sunset till sunrise) per sampling month at each lake from July to October 2011 and April–June 2012 (plus two samplings in May and June 2011). We used an automatic bat recorder, Batcorder (Ecoobs, Nurnberg, Germany), with an omnidirectional microphone, hanging on a 2‐m pole, with the microphone parallel to the ground, placed about 3–4 m from lakeshore.

We used the same mode (“Auto+Timer”) and the same settings of the batcorder (quality: 20; threshold: −27 dB; post‐trigger: 400 ms; critical call frequency: 16 kHz, sample rate: 500 kHz) in all recordings. The distance in which a recorder can record bat calls varies according to species, individuals, habitats, weather conditions (humidity, air temperature), and the recorder's settings and sensitivity. Some species have loud calls, such as *Nyctalus* spp*, Eptesicus* spp. while others have low amplitude calls like *Myotis* spp. or *Plecotus* (though *Plecotus* may be as loud as *Nyctalus* sometimes). In between are *Pipistrellus* species. The batcorder, with the settings we used, can record *Pipistrellus* species up to 10–15 m, *Myotis/Plecotus* species up to 2–10 m and *nyctaloid* species (genera *Nyctalus, Vespertilio, Eptesicus, Tadarida*) up to 20–40 m (pers. comm. Volker Runkel, Ecoobs, Germany). Due to the different sensitivity of the recorder for different species, the recordings are not comparable between species, which was not anyway our aim. Bat activity cannot be compared between species in acoustic studies due to differences in species frequency rates and echolocation intensity (Stahlschmidt & Bruhl, 2012 and references therein). However, the recordings even having a possible bias due to differences between call characteristics of the species, they represent the bat activity situation in each location and the recordings of the same species are comparable between seasons and locations.

Usually bats stop hunting when it rains (Mcaney & Fairley, 1988; Roué & Barataud, 1999) and researchers usually record bats when there is no rain, low wind (<10 km/hr), and at least a medium temperature at the sunset (e.g., at least 10°C, Kusch & Idelberger, 2005). We followed these recommendations as much as possible. Bat activity was defined as seconds of recording of bat passes per hour of recording in each night. The time that the recording was stopped due to rain was excluded from the total recording time. We also recorded the wind on a subjective scale from 0 (no wind) to 5 (strong wind) at the time of the sunset till midnight.

### 2.4 Acoustic analysis

For acoustic analysis, we used software (from Ecoobs) that is specific for recordings made with batcorder: bcAdmin for the management of recorded sessions and sequences; bcDiscriminator that recognizes and takes measurements on bat calls in each sequence; and batIdent that uses those measurements to give a potential identification (on a species or group level) with a probability of this identification to be correct. As the above‐mentioned programs do not permit listening to the recordings, the sequences that needed to be manually checked were exported to wav files and opened with Raven Pro (Bioacoustics Research Program 2011). All sequences identified only as “Chiroptera” or “nothing” were checked in Raven Pro. Most could be identified to the species, genus, or group level, few remained as Chiroptera and those that were noise were deleted. The identification was performed by only one of the authors (IS) to avoid bias. For the identification, books (Tupinier, 1997; Barataud, 2002; Koordinationsstellen für Fledermausschutz in Bayern 2009) and papers (Russo & Jones, 2002; Obrist, Boesch, & Fluckiger, 2004) were used. We classified all the calls identified automatically with a probability of ≤70% in the previous lower identification level. The same was true for species, such as *M. alcathoe*, whose presence in the area is unlikely and has not been confirmed before (pers. comm. Wolfgang Fiedler, Max Planck Institute for Ornithology, Germany).

We grouped *Pipistrellus nathusii* and *P. kuhlii*, together, even if they were identified automatically, as due to their similarities in call characteristics, and it is very difficult to distinguish them only from echolocation calls. Nyctaloid species and *Myotis* species were also grouped, respectively, for further analysis, due to their similarities in call characteristics and usually the low probability that BatIdent identifies them. However, for the species list, we used calls that could be with high confidence identified to species level.

Feeding buzzes are sequences where the pulse duration, interpulse intervals, and frequency decrease (Griffin, Webster, & Michael, 1960). They are produced when a bat is hunting an insect. A number of sequences (3,249 sequences, 25% of the total number), randomly selected, covering all recording sessions were checked manually (visually and acoustically), in Raven Pro, for feeding buzzes.

### 2.5 Statistical analysis

To explore the data, the bat activity and insect emergence were plotted per lake fitting generalized additive models (gam) and smooths to the data. We searched for direct relationships, between and within insect emergence and bat activity, using linear regression between total emerged insects (from 5 days and nights preceding the recording night) and night emerged insects; between insects and air temperature at the sunset; between total bat activity and activity of each species/group (*P. pipistrellus, P. pygmaeus, P. nathusii/kuhlii, Myotis* spp., *M. daubentonii* and nyctaloids) with insect samples and between total number of calls and calls with feeding buzzes. For the insect samples, the number of total and night emerged insects and day and night aerial insects were taken to test separately with each of the other parameters. The total and species/group activity was compared among seasons (per lake) and among lakes (all seasons together and per season) using the nonparametric test Kruskal–Wallis. Seasons were considered as following: spring: April and May; summer: June–August and autumn: September and October. To test for differences between day and night aerial insects, we used the Mann–Whitney–Wilcoxon test. To investigate the variables that explain bat activity in each lake (as there was no clear general pattern of bat activity at the three lakes), we applied general linear models (glms, family = Gaussian), with the following explaining variables: insect availability, season, air temperature at the time of sunset and wind. As a measure of insect availability, we used the night aquatic emerged insects (ind per hr per trap). These models were performed separately per lake and for all seasons together. To investigate what influences bat activity on a spatial scale, linear mixed effects models (lmer) were tested with all data together, lake as a random factor and combinations of the same parameters (insects, wind, temperature, Julian day). Then, the data collected in 2012 only were tested in a model that included also the aerial flying insects. The models with the lowest values of AIC were selected (and presented) as those explaining best the variation in the data. All analyses were performed using the statistics package R (R Core 2016) run within R Studio interface, (RStudio 2016). Additional packages that were used were: lme4 (Bates, Maechler, Bolker, & Walker, 2015), pgirmess (Giraudoux, 2014) for Kruskal‐Wallis test, gridExtra (Auguie, 2012), and ggplot2 (Wickham, 2009) for plots and maps, and ggmap (Kahle & Wickham, 2013) for maps.

## 3 RESULTS

### 3.1 Insects

**Figure 2:**
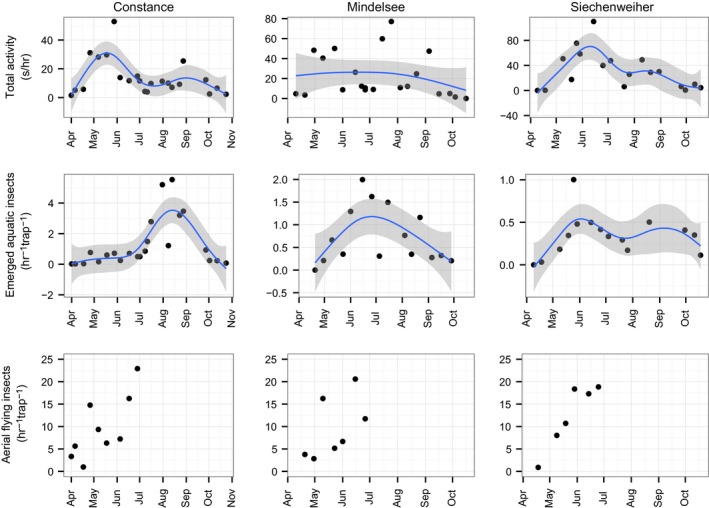
Total bat activity (s/hr of recording) seasonally per lake and night emerged insects per hr per trap (night insects) and aerial flying insects (per hr per trap). A smooth is fitted on the data and the method used is “gam” (generalized additive model). Note the different scales in the plots for bat activity and that the traps used for emerged aquatic and aerial flying insects are different, sample different areas and thus are not comparable. Smoothing was performed with gam method. One outlier for bat activity from Siechenweiher was excluded

The family Chironomidae accounted for the vast majority (82.5%) of the aquatic insects caught in the floating traps during the whole time of their exposure. The night emerged insects were positively related to the total emerged insects at Lake Constance (*R* ^2^ = .771, *F*_1,8_ = 27.05, *p* < .001) and Mindelsee (*R* ^2^ = .817, *F*_1,5_ = 22.25, *p* = .005). The night emerged insects were positive correlated with the water temperature in all lakes (both ln(*x* + 0.1) transformed, *R* ^2^ = .43, *p* < .001). Aquatic insect emergence showed seasonal fluctuations with a peak in August in Lake Constance and in June in Mindelsee and a bimodal pattern with one peak in June and one in September in Siechenweiher (Figure 2).

The most abundant groups of aerial flying insects in all three lakes were Coleoptera (31%) and Chironomidae (16%). Terrestrial origin was attributed to 43% of all aerial flying insects, aquatic (mainly Chironomidae) to 17%, and the rest was not attributed to aquatic or terrestrial origin. This amount might be underestimated as the determination of aquatic or terrestrial origin was rather conservative, and all specimens that were identified only in order or suborder level were characterized as of unknown origin. The day aerial insects (caught during the 3 hr before the sunset) were more in number than the night aerial insects (from sunset to sunrise; *p* < .021 in all lakes).

### 3.2 Bats

We recorded 13 bat species with similar numbers of species at each location (Appendix S1), during 63 nights of recording. We recorded most of the expected species in the region (Appendix S1); however, probably some species could have not been recorded due to their low calls or the height of their flight is too high (e.g., *Pl. auritus*). A greater number of *Myotis* species were recorded at Mindelsee; *Vespertilio murinus* was recorded only at Lake Constance and *Pl. auritus* only at Siechenweiher. The species richness varied seasonally, with the greatest number (12) being recorded in summer and the lowest (7) in autumn. Pipistrelloids accounted for most of the activity (92.3%) in all lakes and seasons. Nyctaloids and *Myotis* spp. contributed very little to the total activity (2.4% and 0.9%, respectively). Mean activity of all bats and of pipistrelloids was highest in Siechenweiher, of *Myotis* spp. in Mindelsee and of nyctaloid in Lake Constance. The species with the highest activity from all lakes during the entire study were *P. pipistrellus* (29.7%), *P. nathusii/kuhlii* (51.3%), and *P. pygmaeus* (2.5%).

A small percentage (4.2%) of the total number of sequences that were checked included feeding buzzes. The number of sequences with feeding buzzes was positively related to the total number of calls checked (*R* ^2^ = .402, *F* = 37.02, *p* < .001), so all the further analysis was performed using all the calls. Positive relationship between feeding buzzes and number of bat passes has been shown elsewhere, confirming that bat passes can be used as a reliable surrogate of foraging activity (Russo & Jones, 2003).

**Figure 3:**
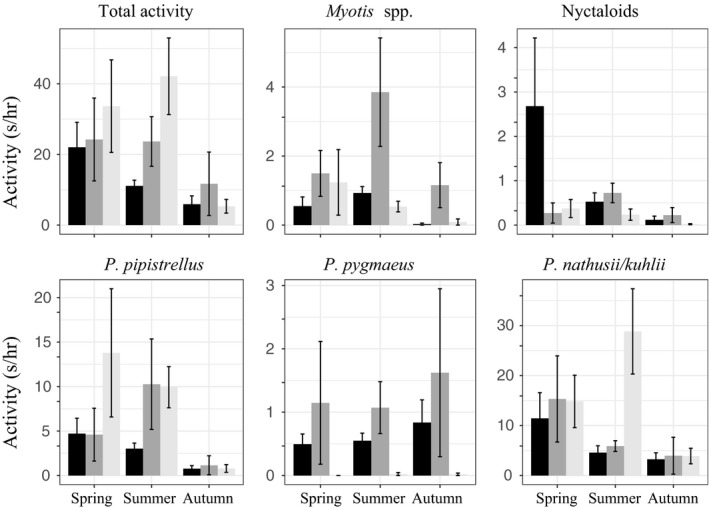
Τotal bat activity and activity per species/group (seconds/hr of recording) per lake and per season. One extreme value from Siechenweiher was excluded as outlier. LG (black): Lake Constance, MI (dark gray): Mindelsee, SI (light gray): Siechenweiher

The total bat activity showed seasonal fluctuations (Figure 2); however, not significant as it was found from the glm models including season or temperature (Table 3). A bimodal pattern of activity was noted in Lake Constance and Siechenweiher with a peak in late spring and early summer respectively and a smaller peak in autumn (Figure 2). In Mindelsee, the pattern seemed less clear and rather unimodal (Figure 2). Differences in activity were noted among lakes and seasons (Figure 3). Significant difference, however, was only noted in the activity of *Myotis* spp. which was greater in summer compared to autumn in Lake Constance (χ^2^(2) = 9.09, *p* = .01).

When comparing bat activity between lakes in the same season, variations in bat activity were more pronounced in summer (Figure 3), when total activity and *P. pipistrellus* activity were greater in Siechenweiher compared to Lake Constance (χ^2^(2) = 8.47, *p* = .014 and χ^2^(2) = 8.07, *p* = .018, respectively); *P. pygmaeus* activity was greater in lakes Mindelsee and Constance compared to Siechenweiher (χ^2^(2) = 12.06, *p* = .002); *P. nathusii/kuhlii* activity was greater in Mindelsee compared to the other two lakes (χ^2^(2) = 12.30, *p* = .002), and *Myotis* spp. activity was greater in Mindelsee compared to Siechenweiher (χ^2^(2) = 6.32, *p* = .042). In spring, only *P. pygmaeus* activity was greater in Lake Constance compared to Siechenweiher (χ^2^(2) = 9.78, *p* = .008; Figure 3), while in autumn there were no significant differences in the bat activity among lakes.

**Figure 4:**
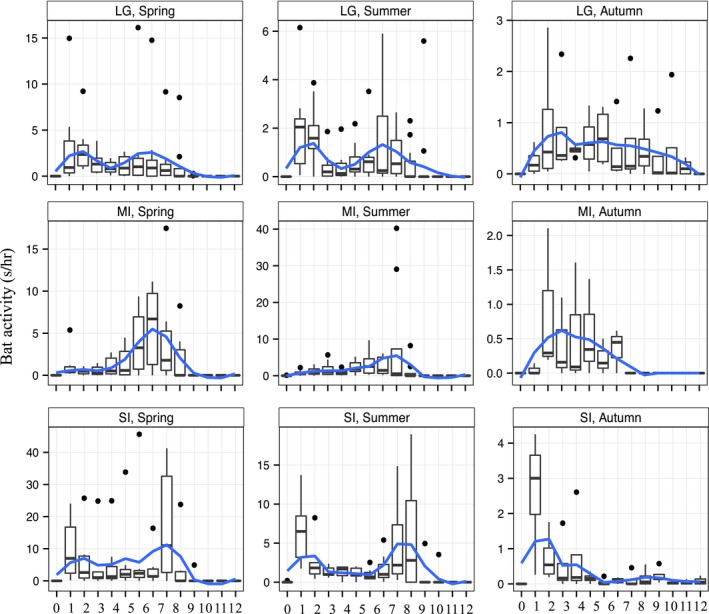
Box‐plots of the hourly total bat activity (s of activity per hr of recording) per lake and per season. Hourly intervals are calculated according to the sunset time. 0: 1 hr before the sunset‐sunset, 1: sunset‐1 hr after sunset, 2: 1–2 hr after sunset, 3: 2–3 hr after sunset and so on. Note the different scales of *Y*‐axis that were kept for better clarity of the hourly pattern although they do not permit easy comparison of the activity. LG, Lake Constance; MI, Mindelsee; SI, Siechenweiher

The bat activity pattern throughout the night also varied both among the lakes and seasons (Figure 4). In Lake Constance, in spring and summer the greatest activity was recorded about 1 hr after sunset with a smaller peak later in the night before sunrise, while in autumn the activity was more evenly distributed throughout the night. In Mindelsee, the peak of activity was in the second part of the night for spring and summer, before sunrise, although there was a considerable activity throughout the night. In Siechenweiher, the activity seemed to be also spread through the night, especially in spring.

### 3.3 Bat activity‐insects

**Figure 5:**
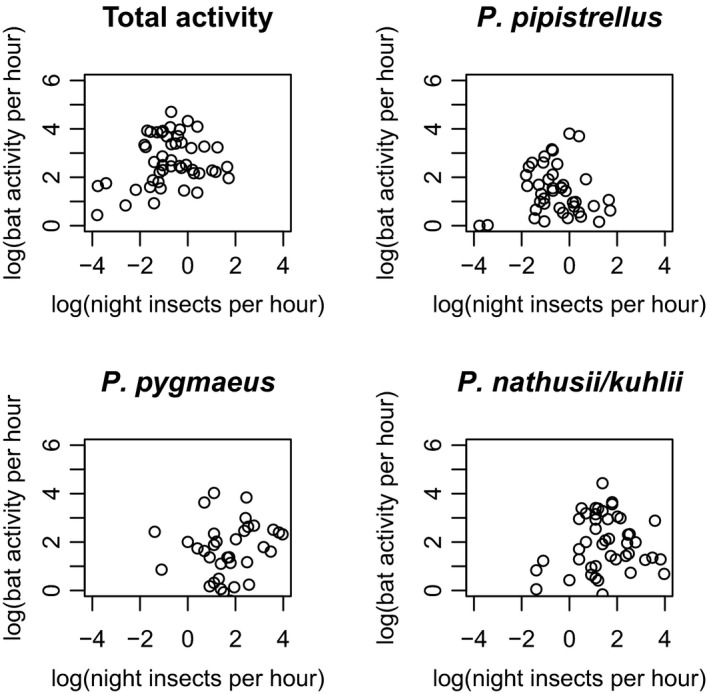
Bat activity (s of activity per hr of recording) related to night insect emergence (number of insects per hr per trap) at all the lakes from: (a) total bat activity, (b) *P. pipistrellus*, (c) *P. pygmaeus* and, (d) *P. nathusii/kuhlii*. Both parameters are ln transformed

Bat activity had weak but positive relationships with the total and night emerged insects (Figure 5; *R* ^2^ = .079, *F*_1,61_ = 5.236, *p* = .026 and *R* ^2^ = .115, *F*_1,52_ = 6.755, *p* = .012, respectively), and both the day and night aerial flying insects (*R* ^2^ = .342, *F*_1,23_ = 11.94, *p* = .002 and *R* ^2^ = .407, *F*_1,23_ = 15.8, *p* = .001, respectively) when all lakes and seasons were considered together (in all cases both bat activity and insects log(*x* + 0.1) transformed. However, this was not always significant when the analysis was performed per lake or per bat species/group (Table 1). Most of the significant relationships were weak (e.g., *P. nathusii/kuhli* and day emerged insects at Siechenweiher, *R* ^2^ = .241, *p* = .033; Table 1), and there was no relationship between bat activity and insects noted in Mindelsee. Only in Siechenweiher, the bat activity correlated with the aquatic insect pattern (Figure 2, Table 1) showing increasing values in spring until they reach a peak in beginning of summer, then they decrease in summer and they increase slightly later in autumn. In spring, in Lake Constance and Siechenweiher the bat activity seemed to increase similarly only with the aerial flying insect numbers (Figure 2, Table 1).

**Table 1:** Statistical significant results (*p* < .05) and *R* ^2^ of the linear regressions between bat activity (seconds of activity per hr of recording) and insects (per hr per trap) per lake. Notice that in Mindelsee, there were no significant relationships found. Both bat activity and insect values are log(*x* + 0.1) transformed

	Lake Constance	Siechenweiher
*P. kuhli/nathusii*—total emerged insects		*R* ^2^ = .241, *p* = .033
*P. kuhli/nathusii*—night emerged insects		*R* ^2^ = .458, *p* = .004
*P. kuhlii/nathussii*—day aerial insects		*R* ^2^ = .924, *p* = .001
*P. kuhlii/nathussii*—night aerial insects	*R* ^2^ = .669, *p* = .004	*R* ^2^ = .528, *p* = .041
*P. pipistrellus*—day aerial insects		*R* ^2^ = .880, *p* = .002
*P. pipistrellus*—night aerial insects	*R* ^2^ = .645, *p* = .005	*R* ^2^ = .612, *p* = .022
*P. pipistrellus*—night emerged insects		*R* ^2^ = .516, *p* = .002
*Myotis* spp.—total emerged insects	*R* ^2^ = .175, *p* = .047	
Nyctaloids—night emerged insects		*R* ^2^ = .325, *p* = .021
Total bat activity—day aerial insects		*R* ^2^ = .916, *p* = .001
Total bat activity—night aerial insects	*R* ^2^ = .749, *p* = .001	*R* ^2^ = .520, *p* = .044
Total bat activity—night emerged insects		*R* ^2^ = .545, *p* = .001

The highest emergence rate was recorded in Lake Constance, then followed Mindelsee and then Siechenweiher, while bat activity was highest in Siechenweiher than in the other two lakes (Figure 2).

The model with lake as random factor explaining best (selected with the AIC criterion) the bat activity and being biological meaningful was the one including emerged aquatic insects, wind speed, and air temperature (Table 2). The best model (selected with the AIC criterion) with lake as random factor for the data of 2012 was the one with explanatory parameters emerged aquatic insects, aerial flying insects, wind, and air temperature. This biologically is the same with the full dataset (the one without aerial flying insects) as the aerial flying insects in a sense contain the aquatic emerged insects (that emerged earlier in the night). When the models were applied for each lake separately for the whole dataset, the models that explained the bat activity variation the best were those with insects (emerged aquatic), wind speed, and temperature in Lake Constance and Siechenweiher and the one with insects, season, and wind in Mindelsee (Table 3). But only insects and wind explained significantly the bat activity only in Lake Constance (Table 3). However, the results of the models should be considered with caution as, based on the diagnostic plots, the models did not seem to fit perfectly to the data, possibly implying that the relationships are complex and more replicates are needed or more parameters to be considered.

**Table 2:** (a) The lmer models (with lake as random factor) that were applied to the full dataset and their Akaike Information Criterion (AIC criterion) value. (b) The results of the best of the above models (the one with the lowest AIC value). Bat activity (s/hr of recording) and insects (emerged night insects per hr) were log*x* + 0.1 transformed

(a)	
Model: bat activity~	AIC
Insects +wind + temperature	161.9
Insects + wind	165.5
Wind + temperature	185.8
Wind + temperature + julday	192.3
Wind + julday	215.0
Insects + wind + temperature + julday	171.7
Insects + wind + julday	192.3
Insects + temperature + julday	194.7
Insects + temperature	190.5

(b) Bat activity~night insects + wind + air temperature + (1|lake)			
Groups	Name	Variance	Std. dev.
Random effects			
Lake	(Intercept)	<0.001	<0.001
Residual		<0.001	<0.001
No. of obs.	47	Groups: Lakes (3)	

	Estimate	Std. error	*t* Value
Fixed effects			
Intercept	0.351	0.887	0.396
Night insects	0.190	0.238	0.798
Wind	−0.267	0.110	−2.432
Temperature	0.165	0.050	3.337

**Table 3:** The general linear models (family = Gaussian) per lake that were selected from those applied to the full dataset according to their Akaike Information Criterion (AIC criterion) value. Bat activity (s/hr of recording) and insects (emerged night insects per hr) were log(*x* + 0.1) transformed

	Estimate	Std. error	*t* Value	*p*(>|*t*|)
Lake Constance Model: bat activity~night insects + wind + temperature				
Intercept	4.382	0.981	4.465	<.001
Night insects	0.905	0.249	3.631	.002
Wind speed	−0.406	0.135	−3.012	.008
Temperature	−0.079	0.057	−1.388	.184
Null Deviance 15.64 on 19 degrees of freedom				
Lake Mindelsee Model: bat activity~ night insects + season + wind				
Intercept	3.063	0.806	3.797	.005
Night insects	0.921	0.488	1.889	.096
Spring	1.157	0.861	1.344	.216
Summer	−0.589	0.824	−0.715	.495
Wind	0.080	0.167	0.476	.647
Null Deviance 12.56 on 12 degrees of freedom				
Siechenweiher Model: bat activity~night insects + wind + temperature				
Intercept	−1.307	3.171	−0.412	.689
Night insects	0.208	1.257	0.165	.872
Wind	−0.283	0.366	−0.774	.457
Temperature	0.275	0.140	1.968	.077
Null deviance 55.99 on 13 degrees of freedom				

## 4 DISCUSSION

### 4.1 Bat activity among lakes

We assessed bat activity using acoustic monitoring, an effective and noninvasive method (e.g., Lintott, Fuentes‐Montemayor, Goulson, & Park, 2013). Simultaneously, we collected, counted, and identified emerging aquatic insects from the lakes and aerial flying insects from the shores where we recorded bat activity. There was no general pattern of bat activity for the studied region, and activity peaks were not necessarily dependent on insect emergence, as predicted. Interestingly, bat activity showed higher spatial than seasonal variability.

Differences in bat activity among the lakes could be related to factors, such as surrounding habitat, proximity to bat roosts and perches, commuting routes, microclimate, and wind exposure. Wind also played a significant role on bat activity, especially in Lake Constance, although we were avoiding recordings in harsh weather conditions. We speculate that waves in Lake Constance, as this place is open and more affected by wind, might explain the low bat activity there. Bats avoid rough surfaces and wavy waters because they interfere with echolocation (Warren, Waters, Altringham, & Bullock, 2000).

Lake size might also explain bat activity differences among the lakes. Although Siechenweiher had low insect emergence per square meter, the small size might have attracted bats from the surrounding area for drinking or feeding. In contrast, a large lake the size of Lake Constance, which had longer lakeshores, could have had lower bat density at the site of our recording location, compared to Siechenweiher. In Mindelsee, which was of intermediate size, higher bat activity was recorded than in Lake Constance, but this could have been due to the availability of habitats. Nevertheless, regardless of the lake size or characteristics, we were able to continuously record considerable bat activity throughout the three seasons and all recording nights in all three study lakes. This confirms the fact that water bodies are important habitats for bats, no matter if it is for feeding or drinking water.

### 4.2 Bat and insect activity

Positive relationships between insects and bat activity were found for Siechenweiher and Lake Constance. The relationships between bat activity and aquatic insect emergence were weak in general, and absent in Mindelsee, indicating that the recorded species might feed partly or not at all on aquatic insects. The stronger relationships that were found between bat activity and aerial flying insects, as in spring at Lake Constance, also imply that bats do not depend only on aquatic insects. The species that are known to feed almost exclusively on aquatic insects, *M. daubentonii* and *P. nathusii* unfortunately could not be discriminated, from congeneric *Myotis* and *P. kuhlii,* respectively, that feed on terrestrial diet. *Pipistrellus pipistrellus*, which showed highest activity, is considered a generalist, while *P. kuhlii* is often associated with aquatic habitats (Vaughan, Jones, & Harris, 1997). Both *P. pygmaeus* and *P. kuhlii* feed on both terrestrial and aquatic insects. Particularly, in Mindelsee, no relationships were found for any of the species/group and insects, implying that insect availability was a poor predictor of bat activity as has been also found elsewhere (e.g., Wolbert, Zenner, & Whidden, 2014, although sampling place was not close to water at all in this study). Nevertheless, in our study we were able to detect some relationships between the emergence of aquatic insects and bat activity.

Indeed, documenting causal relationships requires a more experimental approach, such as by Fukui et al. (2006) who manipulated emerging insect numbers from a river in Japan and showed the relationship between bat activity and aquatic insects. In particular, in the spring bat foraging activity on emerging insects was higher in the control areas than in the treatment where emergence was prevented. In a field study in Sweden, bat activity was better explained by insect availability (that was also mainly Chironomidae) than in our study (DeJong & Ahlén, 1991). In that study, in early spring (May–June), bats were hunted only in woodlands near lakes where aquatic insects were abundant, while insects elsewhere were scarce. Possibly, aquatic insects are more important resources in cases when terrestrial prey is limited. In Germany, food for bats is almost always available, except during hibernation (Zahn, Rodrigues, Rainho, & Palmeirim, 2007) and probably early spring. However, this might not always be the case in Sweden. Other studies have found that bat activity was influenced not only by insect availability but also habitat structure (e.g., in riverine habitats: Hagen & Sabo, 2011), or air temperature (O'Donnell, 2000). Temperature determined if bats fly at all during one night, while invertebrate abundance determined how long they feed (O'Donnell, 2000).

The absence of strong positive relationships between bat activity and emerged aquatic insects is also probably because total activity is not necessarily feeding activity. Echolocation calls were the majority of the recorded sequences, but we also detected social calls and feeding buzzes. Total activity was positively correlated with feeding buzzes, which is true in similar studies (e.g., Rainho, 2007). Thus, total activity is considered a good indication of foraging activity. Feeding buzzes show that bats are following insects, and while the result of the hunt might be unknown, we at least know that bat calls were focusing on insects.

### 4.3 Night pattern bat activity

The nocturnal pattern of bat activity differed between lakes, possibly reflecting differences in habitat, microclimate, and proximity of roosts. The *Pipistrellus* species accounted for most of the activity in all the lakes, and so we do not expect the species composition to be responsible for these activity differences. The nocturnal pattern of bat activity seemed to follow the usual bimodal peaks of insect emergence at dawn and dusk (e.g., Smukalla & Meyer, 1988; Rydell, Entwistle, & Racey, 1996) in the spring/ summer for Lake Constance, and in the summer for Siechenweiher. A possible explanation for the absence of a specific pattern in the autumn could be the low insect availability that might drive bats to search longer for food, or individuals might fly at different hours and places (Swift & Racey, 1983). Seasonal variations in nocturnal activity, foraging time per night, and time of departure and return to the roost have been reported elsewhere as well (Encarnação, Becker, & Ekschmitt, 2010). For example, *P. nathusii* has been reported to show bimodal activity patterns when hunting over wooded sites, while it had unimodal postmidnight activity when hunting in open areas to avoid predators (Ciechanowski, Zajac, Bilas, & Dunajski, 2009).

### 4.4 General conclusions and recommendations

We examined the relationship of aquatic insect emergence to bat activity on a temporal and spatial scale. Our results do not indicate that there is a general pattern applicable for all lakes in the area. However, our data show that, indeed, even small lakes are important for bats as they support diverse bat communities and bat activity (throughout the night and seasons). The relationship between bat activity and insects is not straight‐forward, probably because insect availability is not a limiting factor in the study area. Nevertheless, it is interesting that at the shore of the lake with the lowest aquatic insect emergence, stronger relationship was recorded between the bat activity and the aerial insects than at shores with higher insect emergence, indicating that (probably generalistic) bat species possibly respond to the food availability.

Field experiments, like the one of Fukui et al. (2006) in river, but also in lakes, that control insect availability might provide better insight into what extent aquatic insect resources influence bat activity. Comparative studies in areas with limited water availability might yield insight into the flexibility and resilience of bat species to changing environmental conditions. We showed that although lakes are exporting important amounts of insect biomass to the adjacent terrestrial systems, but may not predict exclusively the behavior of terrestrial consumers.

Korine, Adams, Russo, Fischer‐Phelps, and Jacobs (2016) conclude “studies concerning bats and water are key to better management of water resources.” Therefore, our findings may also be of use for the conservation of bat species and lakes, for example, for taking decisions on small conservation actions (e.g., where to install bat boxes) to which restoration actions should be chosen for a specific water body (e.g., decrease in nutrient levels) or fishing strategies. Data like ours can help to predict possible effects of ecosystem restoration actions on bats, for example, increase in benthivorous fishes in a lake, might decrease emerging insects which can lead to lower insect resources available to bats.

By examining hourly nocturnal activity pattern per season, differences among nights could be masked. In our study area, bats were active throughout the entire night, which was consistent with other studies (e.g., O'Donnell, 2000). If, in places like Mindelsee, monitoring is performed only few hours after sunset, considerable amount of activity will be missed. Therefore, we recommend when nocturnal pattern of bats is unknown, to conduct a pilot study first with a few nights of full recordings in each season and then decide if only few hours of monitoring are enough and when these hours should be.

## CONFLICT OF INTEREST

None declared.

## AUTHORS CONTRIBUTION

IS and KOR conceived and designed the experiments; IS and DG performed the field and laboratory work and analyzed the data; IS drafted the work and wrote the manuscript; IS, DG, and KOR revised the work critically for important intellectual content.
