# Supplementary material for: Seasonal bat activity related to insect emergence at three temperate lakes
Source: Ecol Evol. 2018 Mar 8;8(7):3738–50. doi: 10.1002/ece3.3943 (PMC5901160; doi:10.1002/ece3.3943)
Supplement: Supplementary file 1 [file ECE3-8-3738-s001.docx]

**SUPPLEMENT.—**Bat species reported in the study area and their feeding habits (and references). The study lakes where some species were recorded in the current study are mentioned. Lakes: CO: Constance, MI: Mindelsee, SI: Siechenweiher

| **Species** | **Diet** | **References for diet information** | **present study** | | |  |
| --- | --- | --- | --- | --- | --- | --- |
|  |  |  | **CO** | **MI** | **SI** | **Notes** |
| *Eptesicus serotinus* | Chironomidae, Tipulidae, Lepidoptera, Hemiptera, Hymenoptera, Trichoptera, Arachnida, Coleoptera, Diptera | Vaughan 1997; Gajdosik and Gaisler 2004; Safi and Kerth 2004; Kervyn and Libois 2008 | √ | √ | √ |  |
| *Myotis bechsteinii* | Lepidoptera, Aranaea, Brachycera, Neuroptera, Diptera, Coleoptera, Orthoptera | Vaughan 1997; Safi and Kerth 2004 | √ | √ |  |  |
| *Myotis blythii* | bush crickets, cockhafers, lepidopteran larvae, Orthoptera | Arlettaz 1996; Safi and Kerth 2004 |  |  |  | found in nearby Switzerland |
| *Myotis brandtii/mystacinus* | Diptera, Chironomidae, Simulidae, Tipulidae, Anisopodidae, Culicidae, Arachnida, Lepidoptera, Coleoptera | Vaughan 1997; Safi and Kerth 2004 | √ | √ | √ | *M. brandtii* is reported in the broad region. *M. mystacinus* is most probable in the study area |
| *Myotis daubentonii* | Ceratopogonidae, other Diptera, Trichoptera, Ephemeroptera, Neuroptera, aquatic Diptera, mostly Chironomidae | Vaughan 1997; Swift and Racey 1983; Flavin et al. 2001; Safi and Kerth 2004; Todd and Waters 2007 | √ | √ | √ | aerial hawking above water surface and trawling from water surface |
| *Myotis myotis* | Coleoptera (Carabidae: carabid beetles), Lepidoptera, Orthoptera (Gryllotalpidae: mole crickets) | Arlettaz 1996; Safi and Kerth 2004 |  | √ | √ |  |
| *Myotis nattereri* | Lepidoptera, Dermaptera, Heteroptera, Chironomidae, Aranea, Diptera, Muscidae/Anthomyiidae, Brachycera etc, Coleoptera, Arachnida | Vaughan 1997; Safi and Kerth 2004; Andreas et al. 2012; Hope et al. 2014 |  | √ | √ |  |
| *Nyctalus noctula* | Lepidoptera, Diptera, Coleoptera, Tipulidae, Culicidae, Trichoptera, Chironomidae etc | Gloor et al. 1995; Vaughan 1997; Kanuch et al. 2005 | √ | √ | √ |  |
| *Pipistrellus kuhli* | Culicidae, Lepidoptera, Chironomidae, Ceratopogonidae, Hymenoptera, Brachycera, Tipulidae, Coleoptera | Goiti et al. 2003; Safi and Kerth 2004 | √ | √ | √ |  |
| *Pipistrellus nathusii* | mainly Diptera, Lepidoptera but also Ephemeroptera, Trichoptera, Coleoptera etc, mainly aquatic Diptera Chironomidae | Vaughan 1997; Safi and Kerth 2004; Krüger et al. 2013 | √ | √ | √ |  |
| *Pipistrellus pipistrellus* | mostly Diptera (flies), Trichoptera, Ephemeroptera, Neuroptera, less Coleoptera, Lepidoptera, Diptera, Chironomidae, Ceratopogonidae, Tipulidae, Pscychodidae, Anisopodidae, Empididae | Vaughan 1997; Swift et al. 1985 | √ | √ | √ |  |
| *Pipistrellus pygmaeus* | most prefered prey: small Nematocera (mainly Chironomidae and Ceratopogonidae, Culicidae, Simulidae), also Coleoptera, Trichoptera etc | Bartonička et al. 2008 | √ | √ | √ |  |
| *Plecotus auritus* | mostly Lepidoptera, Coleoptera, Trichoptera, Neuroptera, Brachyptera, Dermaptera, Diptera, Arachnida | Swift and Racey 1983; Vaughan 1997; Safi and Kerth 2004; Andreas et al. 2012 |  |  | √ | identified as *Plecotus* spp. |
| *Vespertilio murinus* | Chironomidae, Diptera, Lepidoptera, Hemiptera | Rydell 1992; Safi and Kerth 2004 | √ |  |  |  |
| *Plecotus austriacus* | Mostly Lepidoptera, Diptera too | Vaughan 1997; Safi and Kerth 2004 |  |  |  |  |
| *Barbastella barbastellus* | Lepidoptera, Diptera, few spiders, plant remains | Vaughan 1997; Safi and Kerth 2004 |  |  |  | found in nearby Switzerland |
| *Rhinolophus ferrumequinum* | Lepidoptera, Coleoptera | Vaughan 1997; Safi and Kerth 2004 |  |  |  | found in nearby Switzerland |
| *Nyctalus leisleri* | mainly Scatophaga stercoraria, Scarabeoidea, Acari but also less Ephemeroptera, Trichoptera, Chironomidae, Ceratopogonidae, Culicidae, mainly Diptera, Muscidae, Tipulidae, Anisopodidae, Coleoptera, Lepidoptera | Vaughan 1997; Shiel et al. 1998; Safi and Kerth 2004; Kanuch et al. 2005 |  |  |  | found in the broad area and in Switzerland |
| **19** |  |  | **10** | **11** | **11** |  |

**References**

Andreas, M., A. Reiter, and P. Benda. 2012. Dietary composition, resource partitioning and trophic niche overlap in three forest foliage-gleaning bats in Central Europe. Acta Chiropterologica 14(2): 335-345.

Arlettaz, R. 1996. Feeding behaviour and foraging strategy of free-living mouse-eared bats, *Myotis myotis* and *Myotis blythii*. Animal Behaviour 51(1): 1-11.

Bartonička, T., Z. Rehak, and M. Andreas. 2008. Diet composition and foraging activity of *Pipistrellus pygmaeus* in a floodplain forest. Biologia 63(2): 266-272.

Flavin, D. A., S. S. Biggane, C. B. Shiel, P. Smiddy, and J. S. Fairley. 2001. Analysis of the diet of Daubenton's bat *Myotis daubentonii* in Ireland. Acta Theriologica 46(1): 43-52.

Gajdošik, M., and J. Gaisler. 2004. Diet of two *Eptesicus* bat species in Moravia (Czech Republic) Folia Zoologica 53(1): 7-16.

Gloor, S., H.-P.B. Stutz, and V. Ziswiler. 1995. Nutritional habits of the noctule bat *Nyctalus noctula* (Schreber, 1774) in Switzerland. Myotis 32-33: 231-242.

Goiti, U., P. Vecin, I. Garin, M. Salona, and J. R. Aihartza. 2003. Diet and prey selection in Kuhl's pipistrelle *Pipistrellus* *kuhlii* (Chiroptera : Vespertilionidae) in south-western Europe. Acta Theriologica 48(4): 457-468.

Hope, P. R., K. Bohmann, M. T. Gilbert, M. L. Zepeda-Mendoza, O. Razgour, and G. Jones. 2014. Second generation sequencing and morphological faecal analysis reveal unexpected foraging behaviour by *Myotis nattereri* (Chiroptera, Vespertilionidae) in winter. Frontiers in Zoology 11: 39.

Kanuch, P., Kristin, A., and J. Kristofik. 2005. Phenology, diet, and ectoparasites of Leisler's bat (*Nyctalus leisleri*) in the Western Carpathians (Slovakia). Acta Chiropterologica 7(2): 249-257.

Kervyn, T., and R. Libois. 2008. The diet of the serotine bat - A comparison between rural and urban environments. Belgian Journal of Zoology 138(1): 41-49.

Krüger, F., E. L. Clare, W. O. C. Symondson, O. Keišs, and G. Pētersons. 2013. Diet of the insectivorous bat *Pipistrellus nathusii* during autumn migration and summer residence. Molecular Ecology 23(15): 3672-3683.

Rydell, J. 1992. The diet of the parti-coloured bat *Vespertilio murinus* in Sweden. Ecography 15: 195-198.

Safi, K., and G. Kerth. 2004. A comparative analysis of specialization and extinction risk in temperate-zone bats. Conservation Biology 18(5): 1293-1303.

Shiel, C. B., P. L. Duverge, P. Smiddy, and J. S. Fairley. 1998. Analysis of the diet of Leisler's bat (*Nyctalus* *leisleri*) in Ireland with some comparative analyses from England and Germany. Journal of Zoology 246: 417-425.

Swift, S. M., P. A. Racey, and M. I. Avery. 1985. Feeding ecology of *Pipistrellus pipistrellus* (Chiroptera, Vespertilionidae) during pregnancy and lactation. 2. Diet. Journal of Animal Ecology 54(1): 217-225.

Swift, S. M., and P. A. Racey. 1983. Resource partitioning in two species of Vespertilionid bats (Chiroptera) occupying the same roost. Journal of Zoology 200(2): 249-259.

Todd, V. L. G., and D. A. Waters. 2007. Strategy-switching in the gaffing bat. Journal of Zoology 273(1): 106-113.

Vaughan, N. 1997. The diets of British bats (Chiroptera). Mammalian Review 27(2): 77-94
